# Supplementary material for: The oncolytic adenovirus TILT-123 with pembrolizumab in platinum resistant or refractory ovarian cancer: the phase 1a PROTA trial
Source: Nat Commun. 2025 Feb 5;16:1381. doi: 10.1038/s41467-025-56482-w (PMC11799410; doi:10.1038/s41467-025-56482-w)
Supplement: Supplementary file 1 — Supplementary Information [file 41467_2025_56482_MOESM1_ESM.pdf]

Supplementary Figures

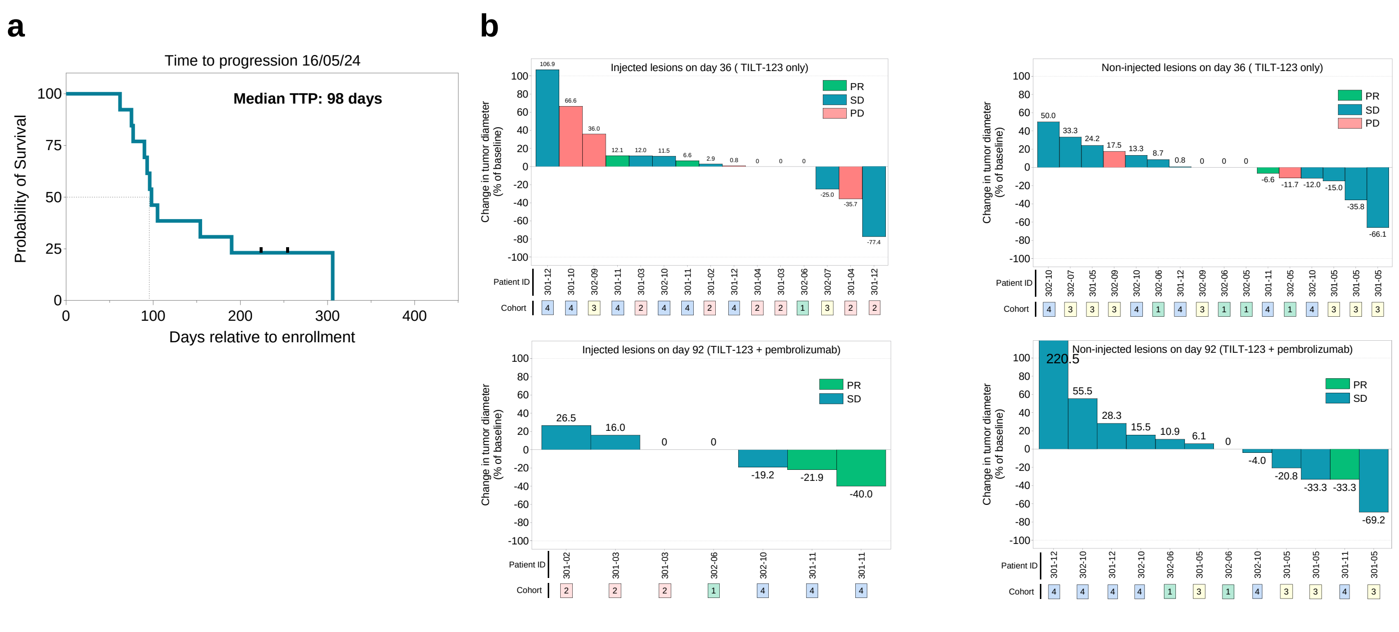

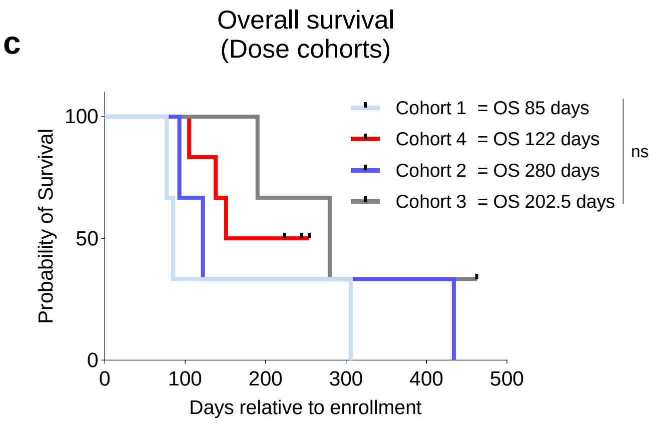

Comparison	p-value (Mantell-Cox Logrank test)
1 vs 2	0.3430
1 vs 3	0.3430
1 vs 4	0.3197
2 vs 3	0.3430
2 vs 4	0.4237
3 vs 4	0.5138

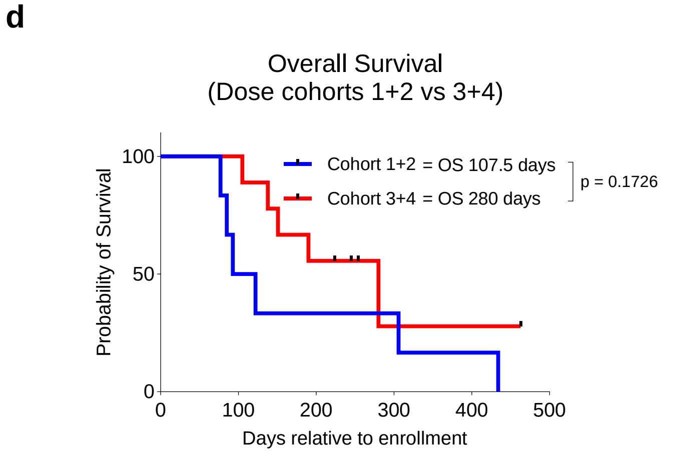

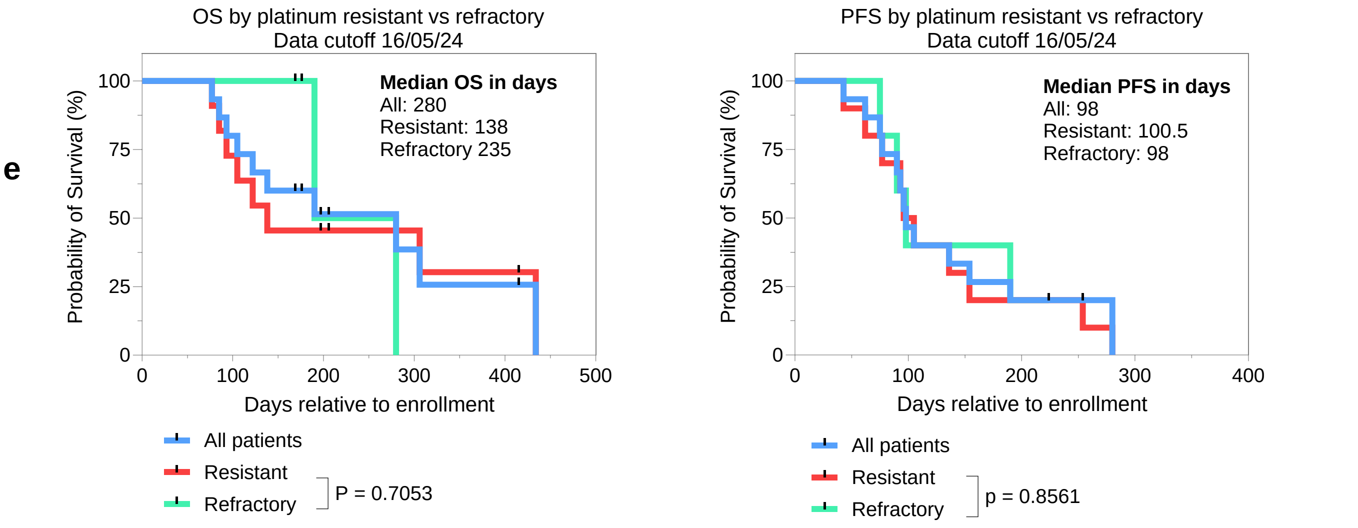

Supplementary figure 1. TTP, individual tumor changes and overall survival analysis by cohort and platinum status. a. Time to progression (TTP). b. Waterfall plot summary of changes in tumor diameter for injected and non-injected target lesions on day 36 and 92. Bars are coloured according to best overall response for the patient with green, teal and red corresponding to partial response, stable disease and progressive disease. c. Comparison of overall survival between dose cohorts 1, 2 3 and 4. d. Comparison of overall survival between lower and higher dose cohorts (1+2 vs 3+4) e. Overall survival (left) and progression free survival (right) by platinum status. Statistical significance of overall survival and progress free survival between groups was calculated using Long-rank (Mantel-Cox) test. n = 15 patients for overall survival and progression free survival analysis. ns = not significant.

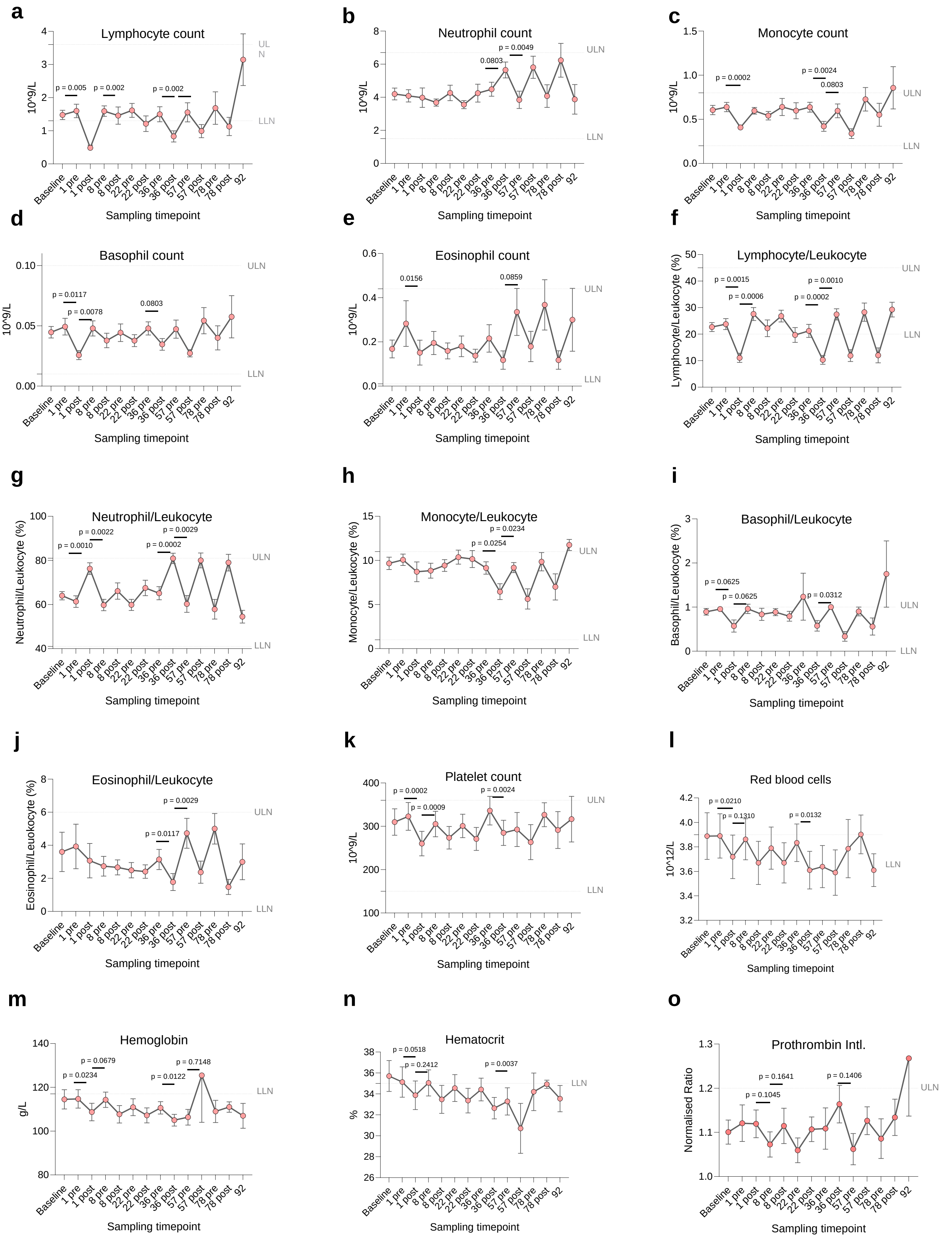

Supplementary figure 2. Summary 1 of clinical blood values during the trial. a. Changes in lymphocyte count. b. neutrophil count. c. monocyte count. d. basophil count. e. eosinophil count. f. lymphocyte/leuokocyte ratio. g. neutrophil/leukocyte ratio. h. monocyte/leukocyte ratio. i. basophil/leukocyte ratio. j. eosinophil/leukocyte ratio. k. platelet count. l. red blood cell count. m. haemoglobin. n. haematocrit. o. prothrombin time/Intl from baseline to day 92 including pre and post dosing on each treatment day. Data presented as mean +/- SEM and p-value using two-tailed Wilcoxon matched-pairs t-test. ULN = upper limit of normal. LLN = lower limit of normal. Exact p values are provided. n = 15 patients.

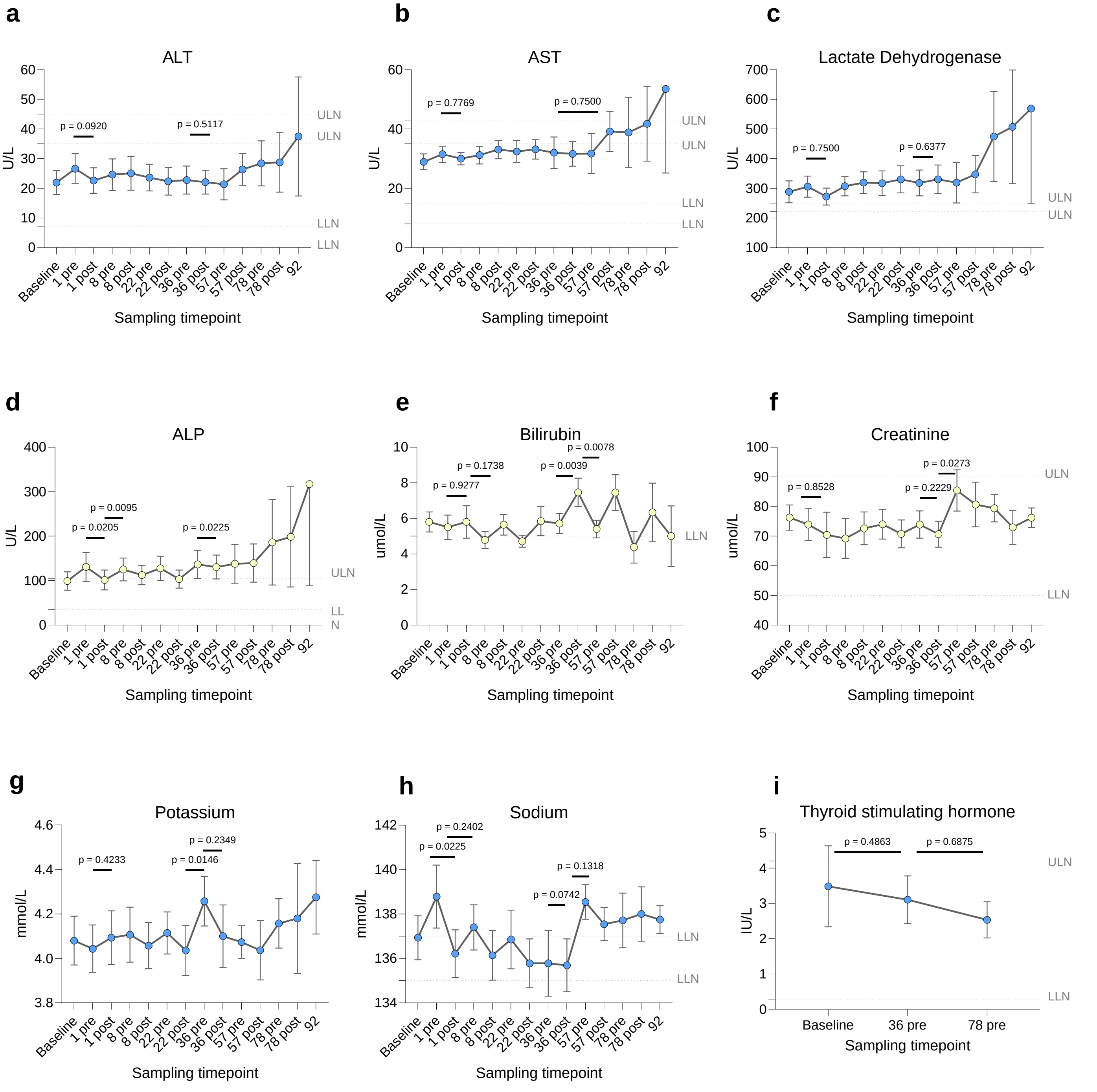

Supplementary figure 3. Summary 2 of clinical blood values during the trial. Changes in ALT (a), AST (b), Lactate dehydrogenase (c), ALP (d), Bilirubin (e), Creatinine (f), Potassium (g), Sodium (h), Thyroid stimulating hormone (i) from baseline to day 92 including pre and post dosing on each treatment day. Data are presented as mean +/- SEM and p-value calculated us two-tailed Wilcoxon matched-pairs t-test. . ULN = upper limit of normal. LLN = lower limit of normal. Exact p values are provided. n = 15 patients.

Patient ID	Cohort	Cancer type	Staging at First Diagnosis at first diagnosis	Primary tumor size and extent at first diagnosis	Regional lymph node involvement at first diagnosis	Distant metastasis at first diagnosis	Laterality	Ascites status
302-05	1	Fallopian tube cancer	IIIB	T3b	NX	M0	Right	Positive
302-06	1	Epithelial ovarian cancer	IIIC	T3c	NX	M0	Bilateral	Negative
301-01	1	Primary peritoneal cancer	IVA	T3c	N0	M1a	Bilateral	Positive
301-02	2	Epithelial ovarian cancer	IIIC	T3a	NX	M0	N/A	Negative
301-03	2	Epithelial ovarian cancer	IIIC	T3a	NX	M0	N/A	Negative
301-04	2	Epithelial ovarian cancer	IVA	T3a	NX	M0	N/A	Negative
302-07	3	Fallopian tube cancer	IVB	T2b	N0	M1a	Left	Negative
301-05	3	Epithelial ovarian cancer	IIIA2	T3a	NX	M0	Bilateral	Negative
302-09	3	Epithelial ovarian cancer	IIIB	T3b	N0	M1a	Bilateral	Negative
302-10	4	Epithelial ovarian cancer	IVB	T2b	N1	M1b	Right	Negative
301-10	4	Fallopian tube cancer	IVB	T3c	NX	M1a	Bilateral	Negative
302-11	4	Primary peritoneal cancer	IVB	T3c	NX	M1a	N/A	Negative
301-11	4	Epithelial ovarian cancer	IVA	T1	N0	M0	Bilateral	Negative
301-12	4	Epithelial ovarian cancer	IIIC	N/A	NX	M1a	N/A	Negative
302-12	4	Primary peritoneal cancer	IVA	T3c	N1	M1a	N/A	Positive

Supplementary Table 1. Patient cancer diagnosis including staging, primary tumor size and extent, regional lymph node involvement, distant metastasis at first diagnosis, cohort, laterality and ascites status.

			Cohort 1			Cohort 2			Cohort 3			Cohort 4					
		ID	302-05	302-06	301-01	301-02	301-03	301-04	302-07	301-05	302-09	302-10	301-10	302-11	301-11	301-12	302-12
	Previous systemic cancer treatments																
	Number of previous systemic treatment lines		7	12	4	3	12	7	5	1	8	11	5	9	2	3	12
Chemotherpy	Cyclophosphamide	1					1										
	Carboplatin + Cyclophosphamide	1		1													
	Carboplatin + Docetaxel	2	1						1								
	Carboplatin + Pegylated Liposomal Doxorubicin	3	1				2		1								1
	Carboplatin + Gemcitabine	2		3			1										
	Carboplatin + Paclitaxel	13	1		2	1	4	2	1	1	3	3	1	2	1		2
	Pegylated liposomal Doxorubicin	5		1							1	1	1				1
	Cisplatin	2									2			1			
	Cisplatin + Paclitaxel	1					1										
	Gemcitabine + Paraplatin	1		1													
	Paclitaxel	2	1									1					1
	Gemcitabine	2										1					1
	Topotecan	3		1								1					1
	Docetaxel	1							1								
	Capecitabine + Oxaliplatin	1													1		
	Doxorubicin + Cisplatin	1												1			
	Paraplatin + Paclitaxel	1		1													
Targeted therapy	Niraparib	5	1				1	1			1						1
	Olaparib	3	1									1				1	
	Letrozole	3		1										1			1
	Monoclonal Antibody	1							1								
	Trametinib	1												1			
	Fulvestrant	2												1			
	Bevacizumab	6		1			1				1	1	1	1			
Oncolytic Virus	MV-NIS	1					1										
Hormonal Therapy	Tamoxifen	1		1													
	Progesterone	1		1													
Combinations	Bevacizumab + Carboplatin + Gemcitabine	2	1													1	
	Bevacizumab + Doxorubicin	1			1												
	Bevacizumab + Pegylated Liposomal Doxorubicin	2				1		1									
	Bevacizumab + Paclitaxel	3			1			1						1			
	Pembrolizumab + Bevacizumab	1						1									
	Carboplatin + Pegylated Liposomal Doxorubin + Bevacizumab	3											2				1
	Carboplatin + Paclitaxel + Bevacizumab	4				1		1								2	
	Other cancer treatments																
	Radiation therapy	4		1			1				1				1		
	Surgery	15	3	2	2	1	4	2	1	1	1	1	2	1	1	1	1
	ICI resist/refract?																
	Yes	7 %						1									
	No (ICI Naive)	93 %	1	1	1	1	1		1	1	1	1	1	1	1	1	1

Supplementary Table 2. Previous lines of cancer treatments per patient.

	Number (%) [max grade] of patients reporting AEs				
Event type	Cohort 1 (*n* = 3)	Cohort 2 (*n* = 3)	Cohort 3 (*n* = 3)	Cohort 4 (*n* = 6)	Number of treatment related adverse events grade ≥ 3
Infection like symptoms					
Fever	0	1 (33%) [1]	2 (66%) [2]	3 (50%) [2]	0
Chills	0	1 (33%) [1]	2 (66%) [1]	2 (33%) [1]	0
Cough	0	0	0	1 (17%) [1]	0
Flu like symptoms	0	0	0	1 (17%) [1]	0
General					
Fatigue	1 (33%) [1]	2 (66%) [2]	1 (33%) [2]	2 (33%) [2]	0
Nausea	1 (33%) [1]	1 (33%) [1]	1 (33%) [1]	3 (50%) [1]	0
Headache	0	0	1 (33%) [1]	2 (33%) [1]	0
Delirium	0	0	0	1 (17%) [3]	1 (7%)
Haematological					
Anaemia	1 (33%) [2]	0	0	1 (17%) [2]	0
Gastrointestinal					
Diarrhoea	1 (33%) [2]	0	2 (66%) [2]	2 (33%) [1]	0
Pyrosis	0	0	0	1 (17%) [1]	0
Vomiting	0	1 (33%) [1]	2 (66%) [1]	0	0
Anorexia	1 (33%) [1]	1 (33%) [1]	1 (33%) [1]	2 (33%) [1]	0
Abdominal discomfort	0	1 (33%) [2]	1 (33%) [1]	0	0
Abdominal pain	0	1 (33%) [1]	1 (33%) [2]	1 (17%) [2]	0
Hemoperitoneum	0	0	0	1 (17%) [3]	1 (7%)
Respiratory				0	
Dyspnoea	0	0	0	2 (33%) [1]	0
Local symptoms					
Infusion site reaction	0	3 (100%) [1]	0	0	0
Alopecia	0	0	1 (33%) [1]	1 (17%) [1]	0
Skin rash	0	0	1 (33%) [1]	0	0
Total	5	12	17	26	2

Supplementary Table 3. Adverse events related to treatment as judged and reported by the investigator, stratified by cohort (dose) and maximum grade reported per patient. Total number of treatment related adverse events more than or equal to grade 3 are included. Data are in n (%) [maximum grade reported]. Events reported as of 16/11//24.

Event type	Grade 1	Grade 2	Grade 3	Grade 4	Grade 5	Total
Infection like symptoms						
Fever	8	4	0	0	0	12
Chills	12	0	0	0	0	12
Cough	1	0	0	0	0	1
Flu like symptoms	2	0	0	0	0	2
General						
Fatigue	8	5	0	0	0	13
Nausea	11	0	0	0	0	11
Headache	4	0	0	0	0	4
Delirium	0	0	1	0	0	1
Malaise	1	0	1	0	0	2
Loss of appetite	1	0	0	0	0	1
Haematological						
Anaemia	2	4	0	0	0	6
Renal & Urological	0	0	0	0	0	
Hydronefrosis	0	0	1	0	0	1
Creatinine increase	1	0	0	0	0	1
Acute renal failure	0	0	1	0	0	1
Urinary tract infection	0	1	0	0	0	1
Gastrointestinal & Hepatobillary						
Diarrhoea	8	3	0	0	0	11
Pyrosis	2	0	0	0	0	2
Vomiting	10	3	0	0	0	13
Anorexia	8	1	0	0	0	9
Abdominal discomfort	4	1	0	0	0	5
Abdominal pain	1	4	0	0	0	5
Hemoperitoneum		0	1	0	0	1
Oral mucositis	1	0	0	0	0	1
GERD	1	0	0	0	0	1
Hypoalbuminemia	0	0	1	0	0	1
Enterocolitis	0	0	1	0	0	1
Colonic fistula	0	0	1	0	0	1
Small bowel occlusion	0	0	1	0	0	1
Paralytic ileus	0	0	1	0	0	1
Respiratory						
Dyspnoea	2	1	1	1	0	5
Pleural effusion	0	1	2	0	0	3
Pneumonia	0	0	1	0	1	2
Cardiovascular						
Arrhythmia	1	0	0	0	0	1
Hypotension	0	1	0	0	0	1
Hypertension	0	1	0	0	0	1
Pericardial effusion	0	0	1	0	0	1
Deep vein thrombosis	0	1				1
Oedema	2	2	0	0	0	4
Muscoloskeletal						
Arthralgia	1	0	0	0	0	1
Leg cramp	1	0	0	0	0	1
Shoulder pain	1	0	0	0	0	1
Pelvic pain	0	1	0	0	0	1
Calf pain	1	0	0	0	0	1
Back pain related to thoracocentesis	1	0	0	0	0	1
Humerus fracture	1	2	1	0	0	4
Neurological						
Neuropathy	1	0	0	0	0	1
Neuralgia	0	0	1	0	0	1
Cancer related						
Disease progression	0	0	0	0	8	8
Pain in tumor	3	0	0	0	0	3
Ascites	0	1	0	0	0	1
Local symptoms						
Infusion site reaction	3	0	0	0	0	3
Alopecia	2	0	0	0	0	2
Dryness of skin	1	0	0	0	0	1
Skin rash	1	1	0	0	0	2
Skin infection (umbilicus)	1	0	0	0	0	1
Stoma site infection	1	0	0	0	0	1
Cellulitis	2	1	0	0	0	3
Pain following biopsy/injection	3	0	0	0	0	3
Miscellaneous						
Covid-19	0	1	0	0	0	1
Infection (NOS)	0	1	0	0	0	1
Sepsis	0	0	1	0	0	1
CRP increase	0	1	0	0	0	1
Dysgeusia	1	0	0	0	0	1
Hypokalemia	1	0	1	0	0	2
Hyponatremia	0	1	2	0	0	3
Total	117	43	21	1	9	191
Total per patient	7.8	2.9	1.4	0.07	0.6	12.7

Supplementary Table 4. All adverse events (AEs) reported by grade. Total event type, total per grade and total grade per patient are included. Data cut-off 16/11/24

T563 Urine Summary			
**Patient ID**	Timepoints analyzed	TILT-123 Negative Samples	TILT-123 Positive Samples
**302-06**	Ext2, 3, 4, 5, 6, 7, 8	14/14	0/14
**301-02**	57, 78, 92	5/5	0/5
**301-03**	36, 57, 78, 92	7/7	0/7
**301-04**	1, 22, 36, 57	8/8	0/8
**301-05**	1, 8, 22, 36, 57, 78, 92, Ext	14/14	0/14
**302-07**	1, 8, 22, 36, 57, 78	12/12	0/12
**302-09**	1, 8, 22, 36, 78	10/10	0/10
**302-10**	1, 8, 22, 36, 78	10/10	0/10
**302-11**	1, 8	4/4	0/4
**302-12**	1, 8	4/4	0/4
**Total**		88 (100%)	0/0%)

T563 Saliva Summary			
**Patient ID**	Timepoints analyzed	TILT-123 Negative Samples	TILT-123 Positive Samples
**302-06**	Ext2, 3, 4, 5, 6, 7, 8	11/11	0/11
**301-02**	57, 78, 92	5/5	0/5
**301-03**	36, 57, 78, 92	7/7	0/7
**301-04**	1, 22, 36, 57	8/8	0/8
**301-05**	1, 8, 22, 36, 57, 78, 92, Ext	14/14	0/14
**302-07**	1, 8, 22, 36, 57, 78	11/11	0/11
**302-09**	1, 8, 22, 36, 78	10/10	0/10
**302-10**	1, 8, 22, 36, 78	9/9	0/9
**302-11**	1, 8	4/4	0/4
**302-12**	1, 8	3/3	0/3
**Total**		82 (100%)	0/0%)

T563 Feces Summary			
**Patient ID**	Timepoints analyzed	TILT-123 Negative Samples	TILT-123 Positive Samples
**301-03**	36, 78, 92	3/3	0/3
**302-07**	1, 36	2/2	0/2
**302-09**	1, 36	2/2	0/2
**302-10**	1, 36	2/2	0/2
**302-11**	1	1/1	0/1
**Total**		10 (100%)	0/0%)

Supplementary Table 5. Urine, saliva and feces shedding results as quantified by qPCR.

a

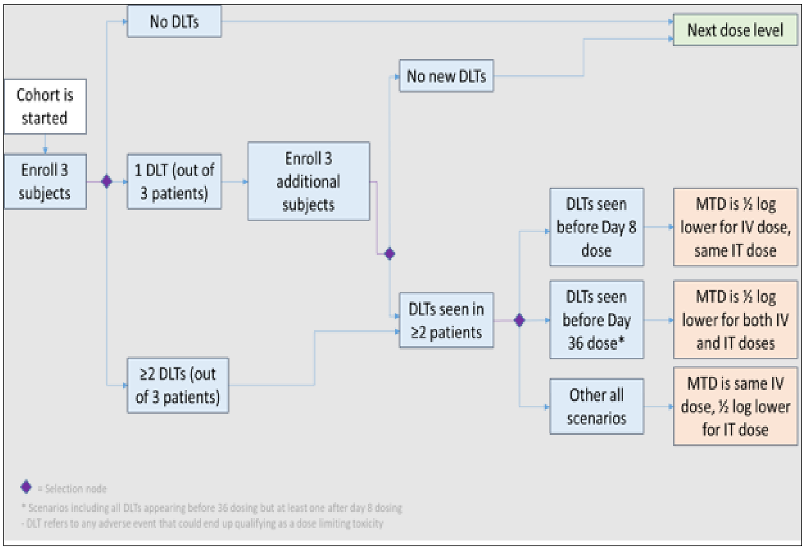

b

1. Grade 4 nonhematologic toxicity (not laboratory).
2. Grade 4 hematologic toxicity lasting ≥7 days, except thrombocytopenia:
   a. Grade 4 thrombocytopenia of any duration
   b. Grade 3 thrombocytopenia associated with clinically significant bleeding
3. Any nonhematologic AE ≥Grade 3 in severity should be considered a DLT, with the following exceptions: Grade 3 fatigue lasting ≤ 3 days; Grade 3 diarrhea, nausea, or vomiting without use of anti-emetics or anti-diarrheals per standard of care; Grade 3 rash without use of corticosteroids or anti-inflammatory agents per standard of care.
4. Any Grade 3 or Grade 4 non-hematologic laboratory value if:
   a. Clinically significant medical intervention is required to treat the participant or
   b. The abnormality leads to hospitalization, or
   c. The abnormality persists for >1 week.
   d. The abnormality results in a Drug induced Liver Injury (DILI)
   e. Exceptions: Clinically non-significant, treatable, or reversible laboratory abnormalities including liver function tests, uric acid, etc.
5. Febrile neutropenia Grade 3 or Grade 4 lasting more than 7 days and with additional presence of clinical symptoms [59]:
   a. Grade 3 is defined as ANC <1000/mm3 with a single temperature of >38.3 degrees C (101 degrees F) or a sustained temperature of ≥38 degrees C (100.4 degrees F) for more than 1 hour
   b. Grade 4 is defined as ANC <1000/mm3 with a single temperature of >38.3 degrees C (101 degrees F) or a sustained temperature of ≥38 degrees C (100.4 degrees F) for more than 1 hour, with life-threatening consequences and urgent intervention indicated.
6. Prolonged delay (>2 weeks) in initiating the second dosage of study-treatment due to treatment-related toxicity.
7. Any treatment-related toxicity that causes the participant to discontinue treatment from day 1 to day 57 (or day 43, for the unacceptable toxicity definition).
8. Missing >25% of TILT-123 doses as a result of drug-related AE(s) during the first cycle.
9. Grade 5 toxicity.
10. A study intervention-related treatment-emergent adverse event (TEAE) that in the opinion of the sponsor and or investigators is of potential clinical significance such that further dose escalation would expose participants to unacceptable risk.

c

The trial investigator will rate the severity of each AE according to the NCI CTCAE guideline version 5.0.
For AEs not possible to capture using the NCI CTCAE, the investigator will use the following definitions to rate the severity of each AE:

- Grade 1 – Mild; asymptomatic or mild symptoms; clinical or diagnostic observations only; intervention not indicated.
- Grade 2 – Moderate; minimal, local or non-invasive intervention indicated; limiting age-appropriate instrumental activities of daily living. Instrumental activities of daily living refer to preparing meals, shopping for groceries or clothes, using the telephone, managing money, etc.
- Grade 3 – Severe or medically significant but not immediately life-threatening; hospitalization or prolongation of hospitalization indicated; disabling; limiting self-care activities of daily living. Self-care activities of daily living refer to bathing, dressing and undressing, feeding self, using the toilet, taking medications, and not bedridden.
- Grade 4 – Life-threatening consequences; urgent intervention indicated. Note, for safety reporting purposes, grade 4 "Life-threatening" is not considered identical to the ICH seriousness criterion "Life-threatening". The investigator must state "life-threatening" as the seriousness criteria on the SAE form in order to be considered life-threatening.
- Grade 5 – Death related to AE.

The grade assigned by the investigator should be the most severe, which occurred during the AE period.

d

- The dosing of this new MTD cohort will be according to the following rules: (1) if both DLT events were seen before day 8, the MTD is half a log (3.3) lower for the i.v. dose but the i.t./i.p. dose remains the same, (2) if both DLT events were seen before day 36 (but both were not seen before day 8), the MTD is half a log (3.3) lower for the both the i.t./i.p. and the i.v. doses, (3) in all other scenarios, the MTD is half a log (3.3) lower for the i.t./i.p. dose while the i.v. dose remains the same.
  Of note, when dose-escalation reaches cohort 4 and a DLT occurs in 2/5 patients at that dose level, an MTD cohort will be according to the following rules: (1) if both DLTs are seen before day 8, the MTD is the cohort 3 i.v. dose and the i.t./i.p. dose remains the same, (2) if both DLTs are seen before day 36, the MTD is the cohort 3 i.t./i.p. and i.v. doses, (3) if both DLTs occur in all other scenarios, the MTD is the cohort 3 i.t./i.p. dose and the i.v. dose remains the same. Pembrolizumab dose will remain the same despite lowering the dose of TILT-123.
- PFS was defined by time from first dose of TILT-123 to the first documented disease progression by CT (RECIST1.1) or death due to any cause, whichever occurs first. If a patient had not had an event (progression or death) before end of trial, then PFS will be censored at the last date known of non-progression.
- TTP was defined by time from first dose of TILT-123 to the first documented disease progression by CT (RECIST1.1) or death. If progression or death was not related to disease then PFS will be censored.

Supplementary figure 4. Clinical protocol extracts. a. Decision tree and DLT implementation for dose-escalation design of trial. b. Trial protocol extract listing toxicities considered as a DLT if occuring during first cycle of treatment with TILT-123 and pembrolizum. c. Trial protocol extract listing definitions to rate severity of each AE if the trial investigator cannot determine using the NCI CTCAE criteria. d. Crtieria used to define MTD, PFS and TTP in the trial.
